# Contrast-enhanced ultrasound for ovary assessment in a murine model: preliminary findings on the protective role of a gonadotropin-releasing hormone analogue from chemotherapy-induced ovarian damage

**DOI:** 10.1186/s41747-018-0076-z

**Published:** 2018-12-19

**Authors:** Massimo Venturini, Alice Bergamini, Laura Perani, Ana Maria Sanchez, Elena Giulia Rossi, Anna Colarieti, Micaela Petrone, Francesco De Cobelli, Alessandro Del Maschio, Paola Viganò, Giorgia Mangili, Massimo Candiani, Carlo Tacchetti, Antonio Esposito

**Affiliations:** 1 Department of Radiology, San Raffaele Scientific Institute, Milan, Italy; 2 Experimental Imaging Center, San Raffaele Scientific Institute, Milan, Italy; 3 Obstetrics and Gynecology Unit, San Raffaele Scientific Institute, Milan, Italy; 4 Vita-Salute San Raffaele University, Milan, Italy

**Keywords:** Disease models (animal), Gonadotropin-releasing hormone, Infertility (female), Ovary, Ultrasonography

## Abstract

The prolonged, gonadotoxic effect of chemotherapy can finally lead to infertility in female cancer survivors. There is controversial evidence regarding the protective role of gonadotropin-releasing hormone analogue (GnRH-a) on chemotherapy-induced ovarian damage. In the present study on a murine model, ultrasound (US) and contrast-enhanced US (CEUS) were firstly used to characterise ovarian glands in normal conditions to validate a preclinical model. In addition, preliminary findings were obtained on anatomical and vascular ovarian changes induced by GnRH-a based on decapeptyl administration. Ovaries were accurately assessed with US and CEUS in a murine model placed in prone position, providing quantitative and reproducible information. Ovaries were identified in 40/40 cases and CEUS analysis was successfully performed in 20/20 cases with 100% technical success. A statistically significant increase of the diameter of the dominant follicle at US and a statistically significant reduced vascularisation at CEUS in decapeptyl-treated mice compared to untreated control mice were recorded. Further studies using US and CEUS in the murine model combining GnRH-a and chemotherapeutic agents will be needed to obtain more translational information useful for clinical practice.

## Key points

- Ovaries, despite their limited size, can be easily identified at US in the murine model as oval, hypoechoic images with net margins, with diameter usually variable from 1 to 2.5 mm, located inferiorly and laterally from the lower pole of the corresponding kidney.
- At US, the diameter of the dominant follicle increased significantly in mice treated with GnRH-a (decapeptyl) mice compared to control untreated mice.
- At CEUS, a reduced vascularisation was found in mice treated with GnRH-a (decapeptyl) compared to control.

## Background

Prognostic improvement obtained in cancer treatment over the last decades emphasises the long-term side effects of chemotherapy. During the last decades, the improvement in prognosis obtained in the treatment of most tumours emphasises the long-term collateral effects of chemotherapy. Gonadotoxicity and fertility reduction are emerging as a crucial challenge for physicians, and this is particularly true in young female cancer survivors [1, 2]. Chemotherapy induces ovarian toxicity through primordial follicular apoptosis, follicular burn-out, stromal fibrosis, and alteration of the vasculature structure [3]. Fertility preservation techniques have become increasingly important to prevent ovarian damage, and the issue of preserving fertility before starting chemotherapy has been addressed by clinical oncologists [1, 4].

Gonadotropin-releasing hormone analogues (GnRH-a) are used in clinical practice, even though the protective mechanisms preventing ovarian insufficiency are still controversial [5]. Translational studies on ovarian glands in mice using ultrasound (US) and contrast-enhanced US (CEUS) have been mainly performed to validate models of ovarian cancer [6], while there is a paucity of experimental studies regarding imaging of normal ovaries. The lack of a well-established US technique for murine ovary detectability and analysis is a gap which has to be filled to build a reproducible preclinical model.

The first aim of this experimental study was to assess the technical aspects and feasibility of US and CEUS to characterise ovaries in normal conditions to validate a preclinical model. The second aim was to obtain preliminary findings on GnRH-a-induced ovarian anatomical and vascular changes assessed by US (dominant follicle diameter) and CEUS (vascular perfusion parameters), respectively.

## Methods

### Study design

This is an experimental, single-centre study in a murine model comparing two groups of female BALB/c mice, a well-known, typical breed of mice used in biomedical research. The mice were treated with subcutaneous injection of a GnRH-a, 100 μL of Decapeptyl SR (Ipsen, Milan, Italy) equivalent to 4.45 mg/kg (*n* = 10, experimental group) or of 100 μL of Dulbecco’s phosphate-buffered saline (Euroclone SPA, Milan, Italy). The two groups were compared to preliminarily assess gonadotropin down-regulation using US and CEUS.

### Mice

The study was approved by the Animal Care and Use Committees of our hospital. Animals were housed in the Institutes’ Animal Care Facilities, according to international standards. BALB/c female mice (14 weeks old) were housed in air-conditioned, pathogen-free, light-controlled animal facilities of the experimental imaging centre of our hospital.

### Animal preparation

Mice were anaesthetised with isoflurane (flurane; Isoba, Schering-Plough, San Diego, CA, USA), 4% in oxygen for induction, 2% for maintenance at a rate of 1 L/min. After the mice were completely anaesthetised, the tail veins were catheterised using 10-cm-long, 27-gauge polyethylene tubing. Mice were positioned in prone position on a MousePad (THM150 MousePad part of the VisualSonics Vevo Integrated Rail System III, Toronto, Canada) equipped with an integrated heater and electrocardiography electrodes. The four legs were secured to electrocardiography pads, with mediation of electrode cream to allow a continuous monitoring of all vital parameters: temperature, respiration rate, cardiac frequency and electrocardiogram. Body temperature was monitored with a rectal probe thermometer. A depilatory cream was used to remove fur from the region of interest, and prewarmed ultrasound gel was used as a coupling agent between the ultrasound probe and the skin.

### US and CEUS examinations

**Fig. 1:**
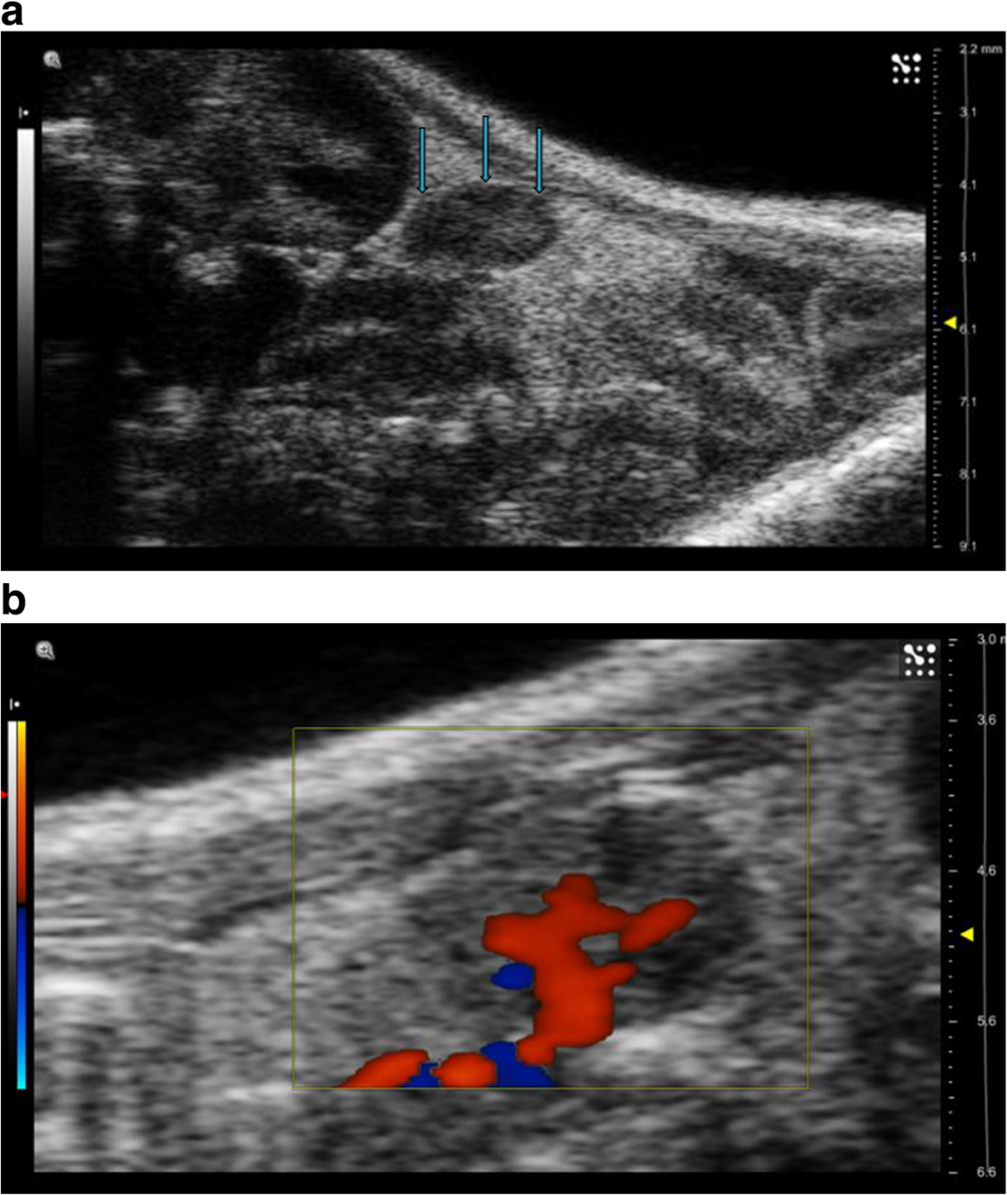
**a** US examination of the mouse placed in prone position shows the right ovary as an oval, hypoechoic nodule with net margin located inferiorly and laterally to the lower pole of the corresponding kidney (*arrows*). **b** Colour Doppler examination well defines vascularisation and particularly the ovarian artery course

B-mode US scans were performed with Vevo 2100 ultrasonographic system high-frequency linear probes, 40 MHz for US and 20 MHz for CEUS (FUJIFILM VisualSonics Inc., Toronto, Canada). All the examinations were performed by the same biologist with more than 5 years of experience in experimental US. All the US and CEUS acquisition data as well as the images were reviewed by a radiologist with more than 5 years of experience in preclinical US and more than 20 years of experience in clinical US and CEUS. Using a mechanical arm that keeps the 40 MHz linear probe fixed, both ovaries were ultrasonically assessed as oval hypoechoic structures, with net margins, vascularised, located inferiorly and laterally from the lower pole of the corresponding kidney (Fig. 1a, b). Ovary major diameter and dominant follicle diameter were major parameters under consideration. After the high-resolution definition of both ovarian glands obtained with the 40 MHz linear probe, the 20 MHz linear probe was mounted on the mechanical arm to perform CEUS, choosing the ovarian gland better identifiable at the US examination. The probe was fixed on the railing system to maintain the acoustic focus at the centre of the ovary at the level of the largest transverse cross section. CEUS studies were performed during intravenous bolus injection of Vevo MicroMarker (Bracco, Geneva, Switzerland). The contrast agent was injected using a syringe pump (Pump 11 Elite, Harvard Apparatus, Holliston, MA, USA) as a bolus via tail vein catheter at a rate of 0.750 mL/min for 5 s (50 μL bolus, 3.5 × 10^7^ microbubbles) in 4 s. During imaging, body temperature was maintained between 36 and 38 °C. The data acquisition was started immediately after contrast agent administration, and cine loops of contrast wash-in were acquired for 55 s. To reduce variability, the used US parameters were the following: power 4%; dynamic range 40 dB; frequency 18 MHz; frame rate 36; contrast gain 49 dB; gate 4.

**Fig. 2:**
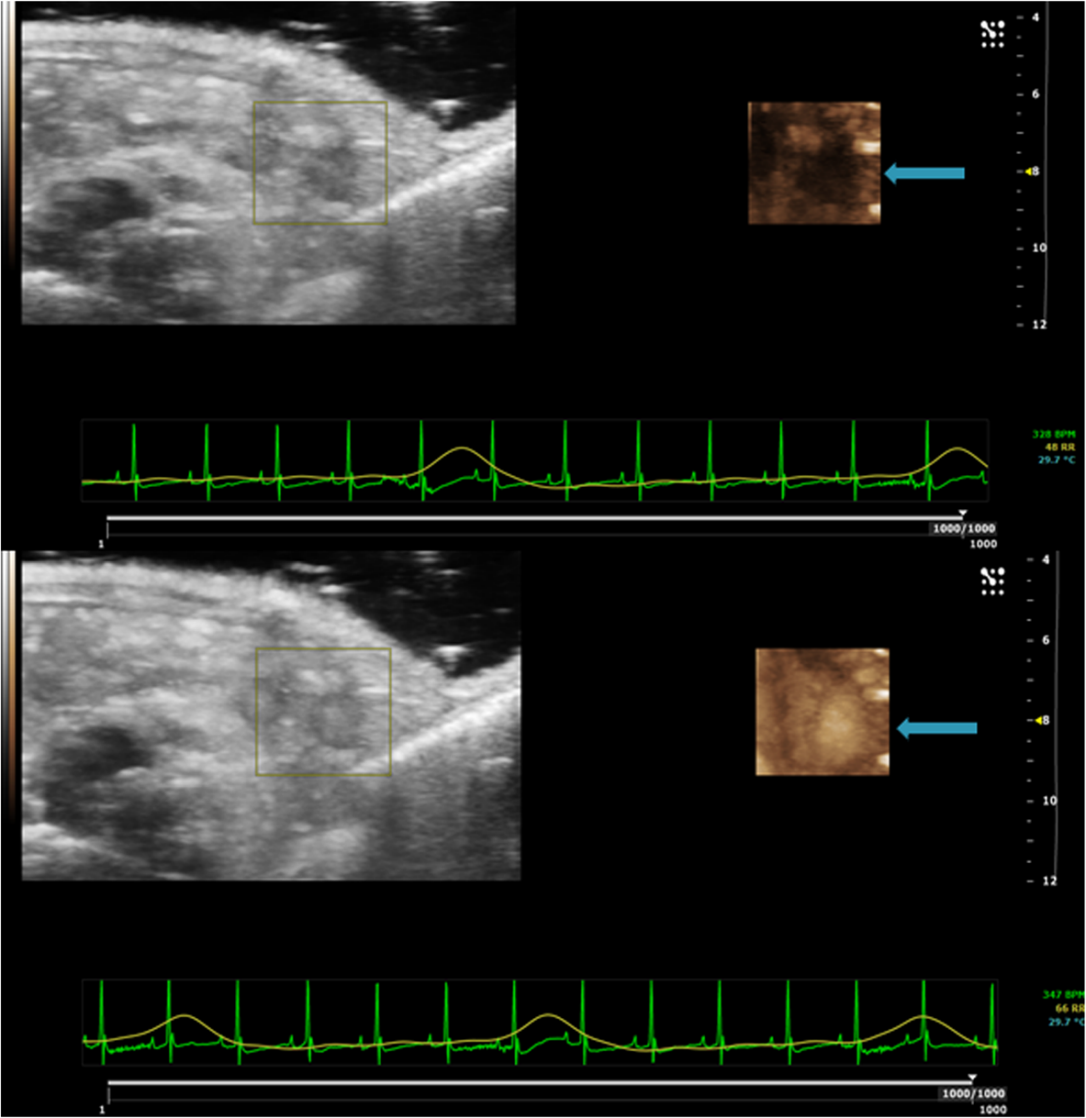
CEUS dynamic acquisition using a side-by-side visualisation (see text) shows the hypoechoic ovary becoming progressively hyperechoic due to the contrast enhancement (*arrows*)

To ensure the quality of the CEUS examination, the ovarian enhancement was assessed during contrast administration, using a side-by-side technique, i.e. in real-time simultaneous visualisation of both the baseline US images demonstrating the ovarian gland as a hypoechoic structure and the CEUS dynamic acquisition showing the ovary progressively becoming hyperechoic (Fig. 2). This assessment was performed at three different time points (day 0, day 10, and day 20).

### CEUS images analysis

Recorded cine loops were processed off-line for time-intensity curve analysis using VevoCQ Advanced Contrast Quantification Software (FUJIFILM VisualSonics). A region of interest was drawn along the perimeter of the ovary. The time-intensity curve for each imaging protocol was plotted, and a mathematical equation model [7] was used to fit the contrast uptake time-intensity curve. Perfusion parameters extracted from the fitted model were the following:

- *Peak enhancement*, defined as the difference between the maximum amplitude in the curve and the baseline level, and is thus indicative of relative blood volume
- *Wash-in rate*, defined as the maximum slope of the fitted curve, in the wash-in phase
- *Time to peak*, which expresses the time in seconds from the time origin to the peak of the fitted curve
- *Rise time*, defined as the time from the instant at which the maximum slope tangent intersects the *x*-axis to the peak of the fitted curve, and is thus independent of the time origin
- *Area under the curve*, defined as area under the curve to infinite time
- *Mean transit time*, defined as the average time required for the contrast agent to pass through the region of interest
- *Perfusion index*, defined as area under the curve divided by mean transit time
- *Wash-in perfusion index*, defined as wash-in area under the curve divided by rise time
- *Wash-in area under the curve*, defined as the area under the curve from starting enhancement to peak enhancement.

### Variable analysis

Technical success, defined as ovaries identification with US and CEUS analysis reliability performed with Advanced Contrast Quantification Software, was preliminarily assessed. Subsequently, comparing decapeptyl-treated mice with untreated mice (controls) at day 0, day 10, and day 20, the following parameters were investigated: ovary major diameter and dominant follicle major diameter (using US); all the perfusion parameters listed in the previous paragraph (using CEUS).

### Statistical analysis

Statistical analysis was performed by *t* test with two-tailed distribution and two-sample unequal variance (GraphPad Prism software, version 5.04). The differences were considered significant when the *p* value was lower than 0.05. Data are presented as means ± standard deviation.

## Results

### Feasibility of US and CEUS

Both ovarian glands were visualised and accurately evaluated at US in all cases at the three different time points. In all cases the ovaries were easily identified at US inferiorly and laterally from the lower pole of the corresponding kidney as oval, hypoechoic nodules with net margins and with a diameter variable from 1 to 2.5 mm. Ovaries were identified in 40/40 cases with 100% technical success at day 0, day 10 and day 20. CEUS analysis with Advanced Contrast Quantification Software was successfully performed in 20/20 cases with 100% technical success at day 0, day 10 and day 20.

### Comparison between decapeptyl-treated mice and control untreated mice

**Fig. 3:**
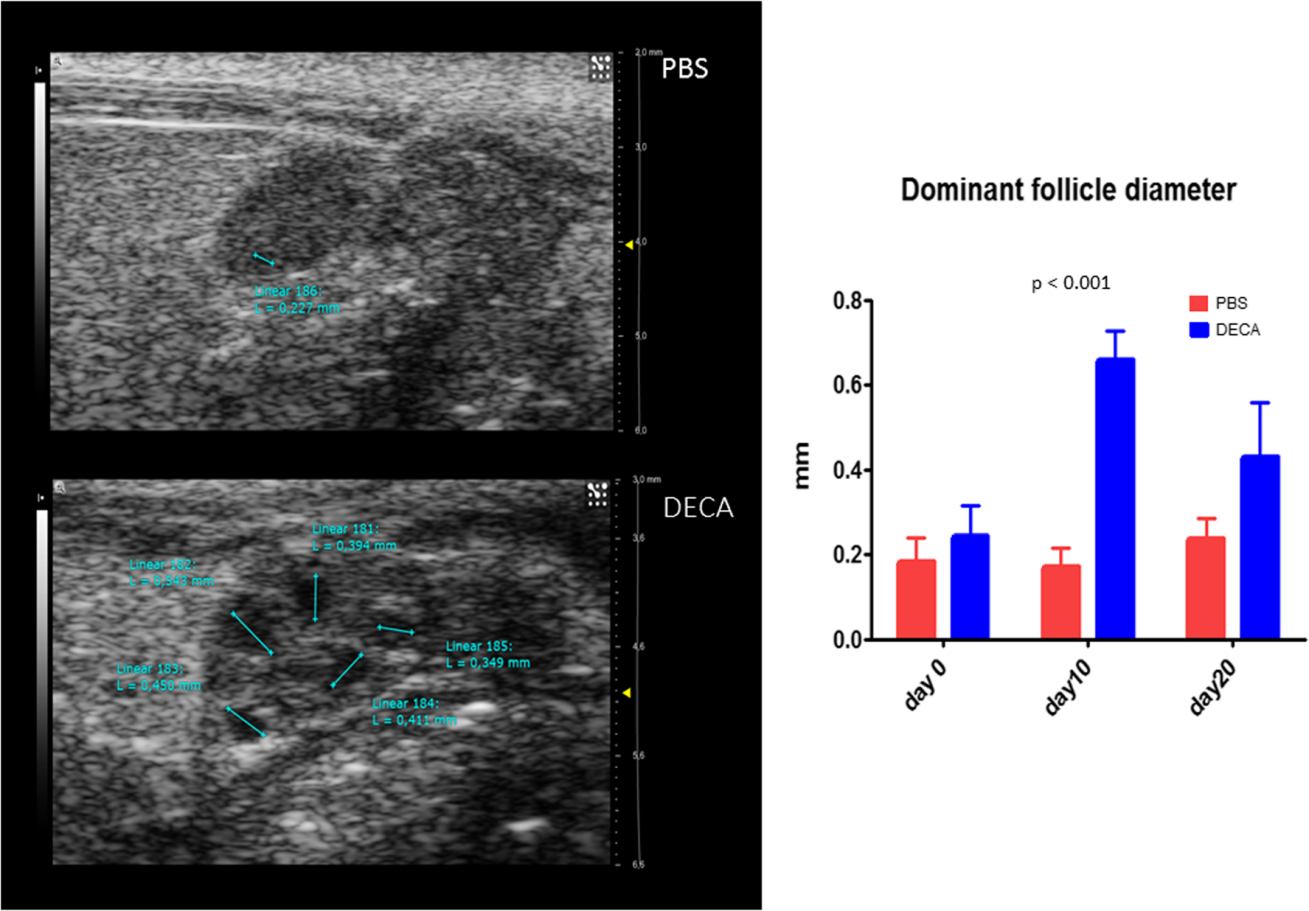
US images (*arrows*) show the increase in the diameter of the dominant follicle in a decapeptyl-treated mouse (*DECA*) compared with a control mouse, which received only phosphate-buffered saline (*PBS*). The graph shows the comparison between the dominant follicle diameter of PBS-treated mice and that of decapeptyl-treated mice

**Fig. 4:**
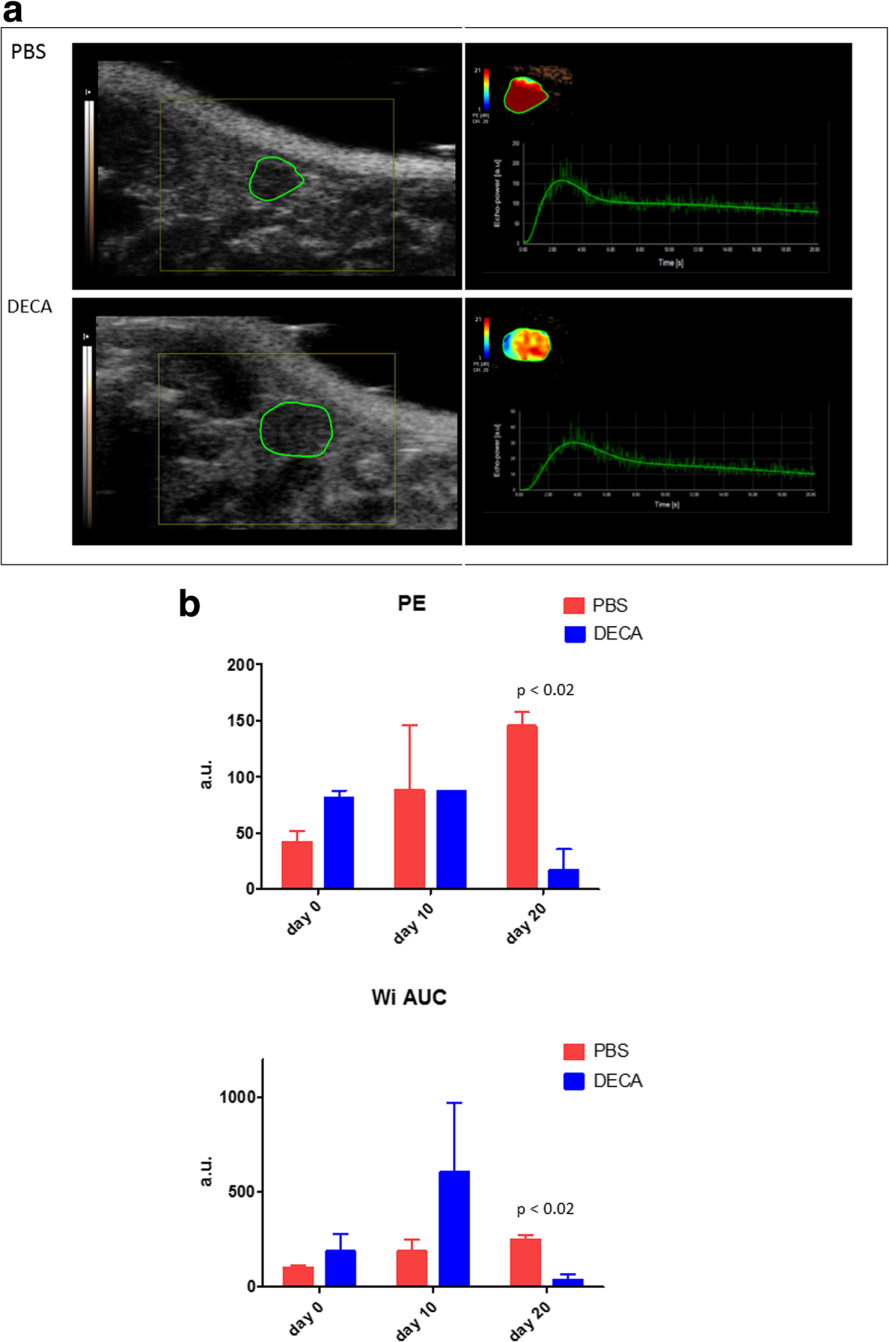
**a** Contrast-enhanced ultrasound shows a reduced ovary perfusion was found in a decapeptyl-treated mouse (*DECA*) compared with a control untreated mouse (*PBS*). For each image, the area circled in *green* shows the ovary. The right side shows the graph representation of the dynamic enhancement. **b** At day 20, significantly reduced peak enhancement (*PE*) and wash-in area under the curve (*WiAUC*) were found in GnRH-a-treated mice (*DECA*) compared with control mice (*PBS*)

Considering US parameters, no significant differences in major diameter were found between decapeptyl-treated mice and controls (Table 1). The diameter of the dominant follicle increased significantly in decapeptyl-treated mice compared to control untreated mice (follicles > 0.4 mm versus follicles < 0.2 mm) at day 10 and day 20 (Table 1, Fig. 3). In the control group no significant variation in this parameter was observed. CEUS of the ovary better identifiable at the preliminary US was successfully performed in all cases, with real-time demonstration of progressive enhancement of the ovarian gland during contrast agent administration. Considering CEUS parameters, a progressive reduction in vascular perfusion was found in decapeptyl-treated mice compared with controls (Fig. 4a). At day 20, a significant reduction in the peak enhancement and wash-in area under the curve was found in decapeptyl-treated mice compared with control untreated mice (Fig. 4b).

**Table 1:** Variation of ultrasound and contrast-enhanced ultrasound parameters of ovaries in decapeptyl-treated mice versus controls

	Day 0			Day 10			Day 20		
	Decapeptyl	Controls	*p* value	Decapeptyl	Controls	*p* value	Decapeptyl	Controls	*p* value
Ultrasound (B-mode)									
Ovary diameter (mm)	2.427 ± 0.70	2.039 ± 0.38	0.190	2.805 ± 0.66	2.260 ± 0.50	0.102	2.470 ± 0.77	2.183 ± 0.48	0.456
Dominant follicle diameter (mm)	0.242 ± 0.07	0.183 ± 0.06	0.248	0.659 ± 0.07	0.170 ± 0.05	< 0.001	0.430 ± 0.13	0.235 ± 0.05	0.070
Contrast-enhanced ultrasound									
Peak enhancement (a.u.)	80.95 ± 6.86	41.8 ± 9.475	0.042	87.25 ± 0.21	88.60 ± 57.56	0.981	17.10 ± 18.67	145.0 ± 12.73	0.015
Wash-in-rate (a.u.)	27.83 ± 15.84	29.67 ± 14.00	0.643	58.87 ± 48.04	66.25 ± 68.94	0.561	9.09 ± 10.35	98.65 ± 28.78	0.054
Time to peak (s)	5.22 ± 2.02	4.07 ± 1.85	0.611	9.94 ± 8.10	4.04 ± 2.00	0.422	4.19 ± 0.67	3.04 ± 0.62	0.219
Rise time (s)	4.27 ± 1.66	3.65 ± 1.58	0.739	9.125 ± 7.66	3.460 ± 1.39	0.412	3.45 ± 0.74	2.545 ± 0.49	0.284
Area under the curve (a.u.)	3145.00 ± 657.61	2120.00 ± 735.39	0.279	5045.00 ± 2538.51	2450.00 ± 1088.94	0.315	362.50 ± 195.87	4975.00 ± 473.76	0.006
Mean transit time (s)	41.76 ± 25.51	59.60 ± 17.34	0.499	76.13 ± 26.51	27.34 ± 0.23	0.121	42.79 ± 33.60	55.82 ± 14.40	0.664
Perfusion index (a.u./s)	86.70 ± 37.19	35.30 ± 1.98	0.190	64.40 ± 10.89	89.35 ± 39.10	0.476	14.84 ± 16.21	91.20 ± 15.27	0.040
Wash-in perfusion index (a.u./s)	54.60 ± 3.82	29.15 ± 7.00	0.457	62.80 ± 4.24	62.25 ± 43.06	0.987	11.35 ± 12.23	97.7 ± 8.91	0.015
Wash-in area under the curve (a.u.)	230.00 ± 73.54	100.65 ± 20.29	0.139	589.50 ± 519.72	185.50 ± 62.93	0.389	34.65 ± 33.87	246.50 ± 24.75	0.019

Data are presented as mean ± standard deviation

*a.u.* arbitrary units

## Discussion

Chemotherapy-induced ovarian damage may be variable, leading to transient/permanent amenorrhea, infertility or early menopause [8, 9]. In the past the use of temporary ovarian suppression with luteinising hormone-releasing hormone analogues [10] and oral contraceptives during chemotherapy has been recommended to preserve fertility [11]. Fertility preservation techniques are becoming a fundamental step of the therapeutic management. Nowadays, embryo/oocyte cryopreservation [12] may be an alternative, although cost and feasibility are unsuitable for all patients. On the other hand, the role of GnRH-a in preventing ovarian damage still remains unclear and controversial.

While single-arm and retrospective studies demonstrated encouraging findings [13, 14], randomised trial data have shown conflicting results [15, 16], partially explained by heterogeneous study populations, variable procedures and different chemotherapeutic treatment regimens.

In this preliminary experimental study, the first end-point was to evaluate with US and CEUS ovaries in normal conditions to obtain a valid preclinical model, and the second end-point was to preliminarily verify the GnRH-a action to perform further prospective studies with chemotherapeutic agents. Ovaries, despite their tiny dimensions (1–2.5 mm), were assessed with US and CEUS: US identification and CEUS analysis of ovaries were successfully performed in all cases (100%, full technical success). Their peculiar position (inferiorly and laterally from the lower pole of the corresponding kidney) and their clear US definition (oval morphology, net margins, hypoechoic structure, vascularisation representation) allowed in all cases a quick identification at US and also an optimal dynamic visualisation during CEUS acquisition. Two other technical issues were important for ensuring US/CEUS feasibility without any problem of acquisition/interpretation data, including perfusion parameter extraction and quantitative software analysis. They were the use of a high-definition US system (Vevo, VisualSonics) equipped with mechanical arm and cutting-edge technology for the study of small experimental animals and the prone position of the mice, eliminating any possible artefacts due to gas bowel. Of note, in one of the few CEUS experimental studies performed to detect doxorubicin-induced damage to gonadal blood vessels, simultaneously performed on ovarian, testicular, and femoral arteries with the mice in conventional supine position [17], ovarian blood flow measurements were affected by bowel movements, probably hampering data findings. In our series performed with mice in prone position, technical problems with US and CEUS were never found. This technical approach can potentially become the standard method to study the ovaries in the murine model, although further studies with a larger cohort of mice will be necessary.

Of nine available CEUS quantitative parameters, the peak enhancement and the wash-in rate showed significant differences between decapeptyl-treated and control mice. From these preliminary findings, decapeptyl-treated mice showed a significant increase in dominant follicle and a significant decrease in vascularisation. These data give new insight in elucidating the potential role of GnRH-a in ovarian protection. GnRH-a administration seems not to impair folliculogenesis, as in treated mice the diameter of the dominant follicle was increased [18]. Interestingly, in our study, the decapeptyl-treated group showed a significant decrease in vasculature compared to controls. Previous studies have described that changes in ovarian blood flow are influenced by gonadotropin secretion, and it has already been reported how the protective of the GnRH-a in mice exposed to the toxic activity of cyclophosphamide could be mediated by a decline in ovarian blood flow and a resulting decrease in the exposure of the ovaries to the chemotherapy agent [19]. Hasky et al. [20] found a decrease in vascular endothelial growth factor in mice treated with doxorubicin and GnRH-a, compared to mice treated with doxorubicin alone.

In conclusion, our preliminary experimental study showed that the ovaries can be assessed with US and CEUS with mice placed in the prone position, providing quantitative information. This technical approach may be the standard method for US evaluation of the ovaries in the murine model. A significant increase in the diameter of the dominant follicle at US and a significant reduction in vascularisation at CEUS in decapeptyl-treated mice compared to controls were observed. This information will be useful for further prospective studies combining GnRH-a and chemotherapy agents evaluating the effects on ovarian function to obtain more translational information useful for clinical practice.

## Abbreviations

CEUS: Contrast-enhanced ultrasound
GnRH-a: Gonadotropin-releasing hormone analogue
US: Ultrasound
